# Physical Characteristics Vary According to Body Mass Index in Japanese Community-Dwelling Elderly Women

**DOI:** 10.3390/geriatrics3040087

**Published:** 2018-11-29

**Authors:** Koji Nonaka, Shin Murata, Kayoko Shiraiwa, Teppei Abiko, Hideki Nakano, Hiroaki Iwase, Koichi Naito, Jun Horie

**Affiliations:** 1 Department of Physical Therapy, Faculty of Health Sciences, Kyoto Tachibana University, Kyoto 607-8175, Japan; murata-s@tachibana-u.ac.jp (S.M.); shiraiwa@tachibana-u.ac.jp (K.S.); abiko@tachibana-u.ac.jp (T.A.); nakano-h@tachibana-u.ac.jp (H.N.); horie-j@tachibana-u.ac.jp (J.H.); 2 Department of Physical Therapy, Faculty of Rehabilitation, Kobe International University, Kobe 658-0032, Japan; iwase@kobe-kiu.ac.jp; 3 Department of Physical Therapy, Hakuho College, Oji 636-0011, Japan; k.naitou@hakuho.ac.jp

**Keywords:** balance, body mass index, elderly, mobility, muscle strength

## Abstract

Background: Body mass index (BMI) is related to health in the elderly. The purpose of this study was to investigate the physical characteristics in underweight, overweight, and obese Japanese community-dwelling elderly women compared to normal-weight elderly women. Methods: The study participants included 212 community-dwelling elderly women. They were categorized as underweight (BMI < 18.5), normal weight (18.5 ≤ BMI ≤ 22.9), overweight (23 ≤ BMI ≤ 24.9), and obese (BMI ≥ 25). Data on skeletal muscle mass index (SMI), number of trunk curl-ups performed within 30 seconds, knee extension strength, one-leg standing time, and walking speed were recorded. Results: In the underweight group, the number of trunk curl-ups was significantly lower than that of the normal-weight group (*p* = 0.011) and the correlation between knee extension strength and walking speed was relatively higher than in the normal-weight group (r = 0.612 vs. r = 0.471). In the overweight group, the SMI was significantly increased (*p* < 0.001), but knee extension strength was not increased (*p* = 0.235) compared to that of the normal-weight group. In the obese group, one-leg standing time was significantly shorter than in the normal-weight group (*p* = 0.016). Conclusions: Physical characteristics vary according to BMI and these findings are useful in assessing and planning interventional programs to improve and maintain physical function in elderly women.

## 1. Introduction

The functions of mobility and balance are decreased in the elderly [1,2]. A slow walking speed, which is one aspect of mobility, is associated with mortality [3]. The risk of falling and developing a subsequent fracture has reportedly been associated with a decline in walking speed [4] and balance [5]. Therefore, it is important for elderly people to improve or maintain mobility and balance. In addition, knowledge of the characteristics of the decline in these functions is necessary for health professionals to assess physical function and to plan intervention programs for the elderly.

Body mass index (BMI) is related to health in the elderly [6], and many studies have investigated the relationship between BMI, mobility, and balance. Walking speed is lower in obese elderly individuals than in normal-weight elderly individuals [6,7]. Likewise, balance ability is reportedly reduced in obese elderly women compared to that of overweight and normal-weight women [8]. Many studies have investigated the relationship between BMI and mobility for individuals who are overweight and obese. Although Woo et al. reported physical function in underweight elderly women [6], there is a paucity of studies in underweight individuals. In addition, some studies have reported no significant differences in walking speed between overweight and normal-weight women [9]. There are thus discrepancies between different studies regarding the relationship between BMI and mobility.

It is useful for health professionals to understand the relationship between BMI, mobility, and balance in order to promote health in the elderly. The purpose of this study was to investigate the physical characteristics, including skeletal muscle mass, muscle strength, balance, and mobility, in underweight, overweight, and obese Japanese community-dwelling elderly women compared with normal-weight elderly women.

## 2. Materials and Methods 

### 2.1. Participants

This was a cross-sectional study involving 212 community-dwelling elderly women. Participants were recruited through public information papers from Yasu city. Criteria for inclusion in the study were as follows: (1) Age more than 60 years, (2) no significant cognitive impairment, and (3) no difficulty walking independently. The exclusion criteria were as follows: (1) Inability to understand the instructions regarding the measurement methods and (2) inability to walk independently. Height and weight of the participants were measured and the BMI was calculated. The participants were grouped into different BMI categories in accordance with Asian criteria to represent underweight (BMI < 18.5), normal weight (18.5 ≤ BMI ≤ 22.9), overweight (23 ≤ BMI ≤ 24.9), and obese (BMI ≥ 25) individuals [6]. In addition, the prevalence of disease was investigated by obtaining information from the participants. The study was conducted in accordance with the Helsinki Declaration. None of the participants had severe cognitive impairment (Mini-Mental State Examination score > 18) and all participants provided written informed consent to participate. This study was approved by the Ethics Committee of the Kyoto Tachibana University (approval number 17-14).

### 2.2. Skeletal Muscle Mass

Skeletal muscle mass was measured by the bioelectrical impedance method using either InBody 430 (Biospace Co., Ltd., Seoul, Korea) or 470 (InBody Japan Inc., Tokyo, Japan). We were able to compare the results of the two devices since both use the same three frequencies (5 kHz, 50 kHz, and 250 kHz). The participants stood on two metallic electrodes and held metallic grip electrodes. The value of the skeletal muscle mass was determined. Skeletal muscle mass was converted into the skeletal muscle mass index (SMI) by dividing the muscle weight by height squared as kg/m^2^. 

### 2.3. The Trunk Curl-Up Test 

The trunk curl-up test was performed as per the method described by Abe et al. [10] to determine trunk function. The participants were placed in the supine position with their knees flexed at 90 degrees and their arms folded across their chest. An examiner held the participant’s knees while she repeated curling up until the elbows touched the thighs and then returned to the initial supine position for 30 s. The score was the number of curl-ups performed correctly in 30 s.

### 2.4. Knee Extension Strength

The knee extension strength was measured as lower extremity muscle strength as per the method described by Bohannon [11]. The participants were asked to sit with their knees and hips flexed at 90°. A hand-held dynamometer (μTas F-1; Anima Corp., Tokyo, Japan) was placed just proximal to the ankle on the anterior surface of the leg and the participant performed maximal isometric muscle contraction. The measurements were performed in both legs with the strongest value used for analysis. 

### 2.5. One-leg Standing Test

To determine the balance ability, one-leg standing time was measured. A one-leg standing test was performed to measure static balance as described by MacRae et al., with a minor modification [12]. In brief, participants were asked to stand with their arms at their sides. The measurement was started when the participant raised a foot off the ground with their eyes open. The measurement was completed if the participant moved their supporting foot or touched the suspended foot to the ground. The maximal time of measurement was set at 120 s. The measurements were performed with both feet and the longest time was used for analysis.

### 2.6. Walking Speed

To assess mobility, walking speed was measured. The participants were asked to walk at their fastest speed in a 10-m walking course, which included a 3-m acceleration and 2-m deceleration zone at each end. The time was recorded for those walking for 5 m of the course. Walking speeds were calculated as m/s.

### 2.7. Statistical Analysis

Comparisons among the four groups were performed using one-way analysis of covariance (ANOVA). The Dunnett post hoc test was used to determine significant differences between the normal-weight group and the other groups when significant differences were observed based on the one-way ANOVA. The chi-square test was performed to compare the prevalence of diseases between groups. Correlations were analyzed using Pearson’s correlation test, which we used to determine correlation coefficients (r). An r value of ≤0.40 was defined as a low correlation, 0.40 < r ≤ 0.60 was a moderate correlation, and 0.60 < r ≤ 0.80 was a high correlation [13]. Participants who were unable to participate in one or more of the tests due to their physical condition were assigned missing values for those tests. Statistical analyses were performed by SPSS ver. 22.0 (IBM Japan, Ltd., Tokyo, Japan) and statistical significance was assumed when *p* < 0.05.

## 3. Results

Characteristics of the participants are shown in Table 1. There were no significant differences in age and height between the groups. Weight and BMI were significantly different between the groups. Besides hypertension, for which a significant difference in the incidence was noted between the groups, the incidence of other diseases was not significantly different between the groups.

A comparison of physical characteristics and correlations between physical characteristics are shown in Table 2 and Table 3, respectively. SMI and trunk curl-ups in the underweight group were significantly lower than in the normal-weight group (*p* < 0.001 and *p* = 0.016, respectively). In the underweight groups, SMI was significantly correlated with trunk curl-ups (r = 0.660; high correlation, *p* = 0.007), and knee extension strength (r = 0.756; high correlation, *p* < 0.001). In addition, walking speed was significantly correlated with trunk curl-ups (r = 0.514; moderate correlation, *p* = 0.020), and knee extension strength (r = 0.612; high correlation, *p* = 0.004). The correlation between knee extension strength and walking speed was stronger than that of the normal-weight group (r = 0.612; high correlation vs. r = 0.471; moderate correlation).

SMI was significantly increased in the overweight group compared to that of the normal-weight group (*p* < 0.001). However, knee extension strength was not increased in comparison to that of the normal-weight group (*p* = 0.235). Knee extension strength was significantly correlated with walking speed (r = 0.435; moderate correlation, *p* = 0.006).

In the obese group, one-leg standing time was significantly less than in the normal-weight group (*p* = 0.016). There was no significant difference in walking speed in the obese and normal-weight groups (*p* > 0.99).

## 4. Discussion

The aim of this study was to investigate the physical characteristics in underweight, overweight, and obese Japanese community-dwelling elderly women compared to normal-weight elderly women. There were several major findings in this study. First, in the underweight group, the number of trunk curl-ups and SMI were decreased and the knee extension strength exhibited a relatively stronger correlation compared to that of the normal-weight group. Second, in the overweight group, the SMI was increased but the muscle strength was not significantly different compared to that of the normal-weight group. Finally, in the obese group, the one-leg standing time was short compared to that of the normal-weight group.

The number of trunk curl-ups in the underweight group was significantly less than that of the normal-weight group. This indicates that underweight elderly women have decreased trunk muscle function. In the underweight group, the number of trunk curl-ups was associated with walking speed. The trunk muscle has been reported to affect trunk control [14], which is important for balance maintenance during walking [15]. Walking speed correlated highly with knee extension strength in the underweight group, while there was only a moderate correlation with knee extension strength in the normal-weight group. In addition, SMI in the underweight group was significantly decreased compared to that of the normal-weight group. Muscle mass has been reported to reflect muscle strength [16]. In this study, SMI was also significantly correlated with trunk curl-ups and knee strength in underweight elderly individuals. The results indicate that the loss of skeletal muscle mass resulted in decreased body weight, which may reflect a decrease in muscle function, particularly in the trunk, thereby affecting mobility.

In the overweight group, the SMI was significantly increased, however, the knee extension strength was not increased compared to that of the normal-weight group. In addition, there was no significant correlation between the SMI and knee extension strength in the overweight group. A positive correlation between skeletal muscle mass and body weight has been reported [17]. In a French study, the appendicular skeletal muscle mass was increased, but the knee extension strength was not increased in obese individuals (BMI > 29) compared to normal-weight individuals (24 < BMI ≤ 29) [18]. These findings are similar to our results. Adipose tissue infiltration into skeletal muscle inhibits the nervous system [19,20] and may produce inflammatory cytokines [21], thereby reducing the muscle force produced. Therefore, lower limb muscle strength may not increase incrementally with skeletal muscle mass because of adipose tissue infiltration into skeletal muscle in the overweight group. Knee extension strength has been associated with maximal walking speed [22]. Similarly, in this study, knee extension strength was associated with walking speed in the overweight group. These findings suggest that muscle strength training is needed to produce muscle power in overweight elderly individuals.

In the obese group, one-leg standing time was significantly shorter than in the normal-weight group. One-leg standing time has been reported to be short in the obese population [23] and our findings concur. One-leg standing was not significantly correlated with trunk curl-ups and knee extension strength. These results suggest that one-leg standing was not affected by muscle strength but by other factors. The moving speed of the center of pressure (CoP) has been reported to be faster in obese than in normal-weight women during one-leg standing [8]. Therefore, obese elderly individuals might be unable to control posture against high speed movement of their CoP, thereby resulting in the shorter one-leg standing time shown in this study. Balance impairment in the elderly is related to the risk of falls and fall-related injuries [24]. Therefore, obese elderly women should perform balance training to decrease fall risk. Walking speed was not significantly different between the obese and normal-weight groups. Furthermore, walking speed has been reported to be unaltered in overweight individuals [9]. This study indicates the possibility that walking speed may not be affected by BMI. Meanwhile, walking speed was slower in the obese (BMI ≥ 25) group than in the normal-weight (18.5 ≤ BMI ≤ 22.9) group [6]. Although the cause of this discrepancy was unclear, it may be that participants in this study were a relatively healthy population. 

There are some limitations to this study. First, this study is cross-sectional in nature, so causality cannot be determined based upon our analysis. Second, the study has a small sample size, particularly for underweight elderly individuals, and third, participants were relatively healthy women. Despite these limitations, our findings provide important information since few studies have investigated the relationship between physical characteristics and BMI, particularly in underweight elderly women.

In conclusion, the physical characteristics vary according to BMI in Japanese community-dwelling elderly women. These findings suggest that interventional programs should be modified according to BMI. For example, trunk function should be improved for underweight individuals, whereas muscle strength may be needed for overweight individuals, and balance training is necessary for obese individuals. Although this study has some limitations, knowledge obtained from this study can be useful for health professionals to plan the assessment of physical function and targeted interventional programs for elderly people.

## Figures and Tables

**Table 1:** Participant characteristics.

	Normal Weight (*n* = 105)	Underweight (*n* = 21)	Overweight (*n* = 41)	Obese (*n* = 45)	F, χ^2^	*p*
Age (years)	73.7 ± 5.8	73.1 ± 6.3	73.9 ± 6.8	73.3 ± 5.1	0.12	0.948
Height (cm)	151.7 ± 5.6	151.3 ± 5.4	151.3 ± 4.8	150.9 ± 4.8	0.26	0.851
Weight (kg)	47.85 ± 4.5	39.5 ± 4.5	54.6 ± 3.8	61.3 ± 5.2	145.44	<0.001
BMI (kg/m^2^)	20.7 ± 1.2	17.2 ± 1.2	23.8 ± 0.5	26.9 ± 1.6	395.07	<0.001
Disease						
Hypertension	41 (39%)	4 (19%)	17 (42%)	26 (58%)	9.531	0.023
Hyperlipidemia	19 (18%)	1 (5%)	4 (10%)	8 (18%)	3.652	0.302
Orthopedic diseases	29 (28%)	7 (33%)	14 (34%)	10 (22%)	1.796	0.616
Stroke	2 (2%)	0 (0%)	1 (2%)	0 (0%)	1.436	0.697
Diabetes mellitus	4 (4%)	0 (0%)	2 (5%)	3 (7%)	1.670	0.664
Rheumatoid arthritis	1 (1%)	1 (5%)	0 (0%)	1 (2%)	2.646	0.449
Cardiovascular disease	5 (5%)	3 (14%)	1 (2%)	2 (4%)	4.252	0.235
Pulmonary disease	3 (3%)	0 (0%)	1 (2%)	1 (2%)	0.625	0.891
Renal disease	3 (3%)	0 (0%)	0 (0%)	0 (0%)	3.101	0.376
Cancer	2 (2%)	0 (0%)	1 (2%)	0 (0%)	1.436	0.697
Others	13 (12%)	6 (29%)	8 (20%)	9 (20%)	4.054	0.256

Values are presented as means ± SD. BMI, body mass index; F, *F*-value; χ^2^, chi-square value

**Table 2:** Comparison of physical characteristics between the normal-weight group and the underweight, overweight, and obese groups.

		Normal Weight (*n* = 105)	Underweight (*n* = 21)	Overweight (*n* = 41)	Obese (*n* = 45)	F	*p*
Skeletal muscle mass	SMI (kg/m^2^)	7.9 ± 0.6	7.1 ± 0.8 ***	8.5 ± 0.6 ***	9.0 ± 0.7 ***	46.935	<0.001
Muscle function	Trunk curl-up (repetition/30 sec)	9.1 ± 6.2	4.7 ± 4.9 *	8.6 ± 6.7	7.0 ± 5.3	3.420	0.018
	Knee extension strength (N)	202.6 ± 51.0	175.5 ± 43.6	219.7 ± 64.6	225.6 ± 50.5	5.002	0.002
Balance	One-leg standing (sec)	40.0 ± 38.8	35.8 ± 38.3	27.2 ± 24.8	23.0 ± 24.1 *	3.247	0.023
Mobility	Walking speed (m/sec)	1.9 ± 0.3	2.0 ± 0.3	1.9 ± 0.3	1.9 ± 0.2	0.735	0.532

Values are presented as means ± SD. BMI, body mass index; SMI, skeletal muscle index; F, *F*-value * *p* < 0.05 and *** *p* < 0.001 vs. the normal-weight group.

**Table 3:** Correlation between the physical characteristics in each BMI group.

		Normal weight	Underweight	Overweight	Obese
SMI	Trunk curl-up	0.348 **	0.600 **	0.285	−0.037
	Knee extension strength	0.295	0.756 **	0.229	0.234
One-leg standing	SMI	0.054	0.022	0.088	0.334 *
	Trunk curl-up	0.263 *	0.372	0.057	−0.021
	Knee extension strength	0.448 ***	−0.088	0.317*	−0.22
Walking speed	SMI	0.148	0.386	0.106	0.035
	Trunk curl-up	0.265 *	0.514 *	0.285	−0.083
	Knee extension strength	0.471 ***	0.612 **	0.435 **	0.368 *
	One-leg standing	0.485 ***	0.245	0.573 ***	0.174

Values are correlation coefficients (r). BMI, body mass index; SMI, skeletal muscle mass index * *p* < 0.05, ** *p* < 0.01, and *** *p* < 0.001 vs. the normal-weight group.

## References

[1] Balogun J.A., Akindele K.A., Nihinlola J.O., Marzouk D.K. (1994). Age-related changes in balance performance. Disabil. Rehabil..

[2] Lauretani F., Russo C.R., Bandinelli S., Bartali B., Cavazzini C., Di Iorio A., Corsi A.M., Rantanen T., Guralnik J.M., Ferrucci L. (2003). Age-associated changes in skeletal muscles and their effect on mobility: An operational diagnosis of sarcopenia. J. Appl. Physiol..

[3] Cesari M., Pahor M., Lauretani F., Zamboni V., Bandinelli S., Bernabei R., Guralnik J.M., Ferrucci L. (2009). Skeletal muscle and mortality results from the InCHIANTI study. J. Gerontol. A Biol. Sci. Med. Sci..

[4] Studenski S., Perera S., Wallace D., Chandler J.M., Duncan P.W., Rooney E., Fox M., Guralnik J.M. (2003). Physical performance measures in the clinical setting. J. Am. Geriatr. Soc..

[5] Thrane G., Joakimsen R.M., Thornquist E. (2007). The association between timed up and go test and history of falls: the Tromsø study. BMC Geriatr..

[6] Woo J., Leung J., Kwok T. (2007). BMI, body composition, and physical functioning in older adults. Obesity (Silver Spring).

[7] Visser M., Harris T.B., Langlois J., Hannan M.T., Roubenoff R., Felson D.T., Wilson P.W., Kiel D.P. (1998). Body fat and skeletal muscle mass in relation to physical disability in very old men and women of the Framingham Heart Study. J. Gerontol. A Biol. Sci. Med. Sci..

[8] Dutil M., Handrigan G.A., Corbeil P., Cantin V., Simoneau M., Teasdale N., Hue O. (2013). The impact of obesity on balance control in community-dwelling older women. Age (Omaha).

[9] Ferreira R.S., Da Silva Coqueiro R., Barbosa A.R., Pinheiro P.A., Fernandes M.H. (2013). Relationship between BMI and physical performance among older adults. Geriatr. Nurs..

[10] Abe T., Yaginuma Y., Fujita E., Thiebaud R.S., Kawanishi M., Akamine T. (2016). Associations of sit-up ability with sarcopenia classification measures in Japanese older women. Interv. Med. Appl. Sci..

[11] Bohannon R.W. (1986). Test-retest reliability of hand-held dynamometry during a single session of strength assessment. Phys. Ther..

[12] MacRae P.G., Lacourse M., Moldavon R. (1992). Physical performance measures that predict faller status in community-dwelling older adults. J. Orthop. Sport. Phys. Ther..

[13] Van Dijk M.M., Meyer S., Sandstad S., Wiskerke E., Thuwis R., Vandekerckhove C., Myny C., Ghosh N., Beyens H., Dejaeger E. (2017). A cross-sectional study comparing lateral and diagonal maximum weight shift in people with stroke and healthy controls and the correlation with balance, gait and fear of falling. PLoS ONE.

[14] Suri P., Kiely D.K., Leveille S.G., Frontera W.R., Bean J.F. (2009). Trunk muscle attributes are associated with balance and mobility in older adults: A pilot study. PM&R.

[15] Helbostad J.L., Moe-Nilssen R. (2003). The effect of gait speed on lateral balance control during walking in healthy elderly. Gait Posture..

[16] Landers K., Hunter G., Wetzstein C., Bamman M., Weinsier R. (2001). The interrelationship among muscle mass, strength, and the ability to perform physical tasks of daily living in younger and older women. J. Gerontol. A Biol. Sci. Med. Sci..

[17] Janssen I., Heymsfield S.B., Wang Z., Ross R. (2014). Skeletal muscle mass and distribution in 468 men and women aged 18−88 yr. J. Appl. Physiol..

[18] Rolland Y., Lauwers-Cances V., Pahor M., Fillaux J., Grandjean H., Vellas B. (2004). Muscle strength in obese elderly women: effect of recreational physical activity in a cross-sectional study. Am. J. Clin. Nutr..

[19] Marcus R.L., Addison O., Dibble L.E., Foreman K.B., Morrell G., Lastayo P. (2012). Intramuscular adipose tissue, sarcopenia, and mobility function in older individuals. J. Aging Res..

[20] Yoshida Y., Marcus R.L., Lastayo P.C. (2012). Intramuscular adipose tissue and central activation in older adults. Muscle Nerve.

[21] Hardin B.J., Campbell K.S., Smith J.D., Arbogast S., Smith J., Moylan J.S., Reid M.B. (2008). TNF-α acts via TNFR1 and muscle-derived oxidants to depress myofibrillar force in murine skeletal muscle. J. Appl. Physiol..

[22] Bohannon R.W. (1997). Comfortable and maximum walking speed of adults aged 20–79 years: Reference values and determinants. Age Ageing.

[23] Villareal D.T., Banks M., Siener C., Sinacore D.R., Klein S. (2004). Physical frailty and body composition in obese elderly men and women. Obes. Res..

[24] Vellas B.J., Wayne S.J., Romero L., Baumgartner R.N., Rubenstein L.Z., Garry P.J. (1997). One-leg balance is an important predictor of injurious falls in older persons. J. Am. Geriatr. Soc..

